# Study protocol: diagnostic accuracy study comparing Cy-Tb and STANDARD F TB-Feron FIA tests for tuberculosis infection diagnosis in Vietnam

**DOI:** 10.1136/bmjopen-2024-085614

**Published:** 2024-08-09

**Authors:** Han Thi Nguyen, Luan Nguyen Quang Vo, Andrew Codlin, Rachel Forse, Tom Wingfield, Kristi Sidney, Emily Lai-Ho MacLean, Jacob Creswell, Beatrice Kirubi, Lina Davies Forsman

**Affiliations:** 1 Department of Medicine, Unit of Infectious Disease, Karolinska Institute, Stockholm, Sweden; 2 Friends for International Tuberculosis Relief, Ha Noi, Vietnam; 3 Friends for International TB Relief (FIT), Ho Chi Minh City, Vietnam; 4 Department of Global Public Health, Karolinska Institute, Stockholm, Sweden; 5 Department of Global Public Health, The Health and Social Protection Action Research & Knowledge Sharing Network (SPARKS), Karolinska Institutet, Stockholm, Sweden; 6 Department of International Public Health and Clinical Sciences, Liverpool School of Tropical Medicine, Liverpool, UK; 7 The University of Sydney, Sydney, NSW, Australia; 8 Stop TB Partnership, Geneva, Switzerland; 9 Department of Infectious Disease, Karolinska University Hospital Solna, Stockholm, Sweden

**Keywords:** Public health, Epidemiology, Diagnostic microbiology, Tuberculosis, Respiratory infections, Tropical medicine

## Abstract

**Introduction:**

The large reservoir of tuberculosis (TB) infections is one of the main reasons for the persistent incidence of TB. Accurate diagnostic tests are crucial to correctly identify and treat people with TB infection, which is vital to eliminate TB globally. The rdESAT-6 and rCFP-10 (Cy-Tb) injection (‘Cy-Tb’), a TB-specific antigen skin test and STANDARD F TB-Feron FIA (‘Standard F TB’) measuring interferon-gamma by fluorescence immunoassay assay are two novel tools for the diagnosis of TB infection which offer advantages compared with current tests in low-resource settings and reduced costs to both health systems and TB-affected people. The proposed study aims to evaluate the diagnostic accuracy of these two new tests for TB infection diagnosis.

**Methods and analysis:**

This cross-sectional study aims to assess the diagnostic accuracy for TB infection of the Cy-Tb skin test and Standard F TB assay (investigational tests) compared with the QuantiFERON-TB Gold Plus (QFT-Plus) assay as the immunological reference standard. Three different cohorts of study participants will be recruited at the Vietnam National Lung Hospital: adults with bacteriologically confirmed pulmonary TB (n=100), household contacts of people with TB (n=200) and people without TB infection (n=50). All consenting participants will undergo simultaneous testing with Cy-Tb, Standard F TB and QFT-Plus. The primary endpoint is the diagnostic accuracy of the Cy-Tb skin test and Standard F TB assay, expressed as sensitivity and specificity against the reference standard.

**Ethics and dissemination:**

Ethical approval was granted by the Vietnam National Lung Hospital Institutional Review Board (65/23/CN-HDDD-BVPTU) and the Swedish Ethical Review Authority (Dnr 2023-04271-01). Study results will be disseminated to the scientific community and policymakers through scientific publications.

**Trial registration number:**

NCT06221735.

**STRENGTHS AND LIMITATIONS OF THIS STUDY**

- This is the first study to compare the accuracy of STANDARD F TB-Feron FIA (Standard F TB) versus QuantiFERON TB Gold Plus (QFT-Plus) as well as the first study to simultaneously compare both Cy-Tb and Standard F TB to QFT-Plus.
- The study will be conducted in one of the 30 countries with the highest tuberculosis (TB) burden in the world, where the tests have not been evaluated before, ensuring the feasibility of recruiting an adequate number of participants.
- All study tests will be quality assured as they will be conducted at the leading microbiological laboratory in Vietnam, the Vietnam National Reference Lab.
- A limitation is that the reference standard used in the study (QFT-Plus assay) also has suboptimal sensitivity, but is currently the best available test for the diagnosis of TB infection and is recommended by the WHO.
- A limitation of the study is its sole performance in one setting, where participants are homogeneous concerning genetic polymorphism.

## Introduction

### Background

 Tuberculosis (TB) is an infectious disease that is a leading cause of morbidity and mortality globally. There were an estimated 1.3 million total deaths caused by TB in 2022.^1^ Vietnam is one of the 30 highest TB burden countries, with an estimated 172 000 persons developing TB (including people living with HIV) in 2022.^2^

TB infection continues to be a significant cause of the increasing number of overall infections. After exposure to *Mycobacterium tuberculosis* (MTB), 5%–10% of people with TB infection will develop active TB, usually within the first 2 years after exposure.^3^ In 2018, the WHO estimated the global prevalence of TB infection to be 23%, meaning approximately 1.7 billion people were infected worldwide.^4 5^ A recent re-estimation using mathematical modelling demonstrated that in order to end TB by 2050, at least one-quarter of the global population living with TB infection would require TB preventive therapy.^6^

The tuberculin skin test (TST) and interferon-gamma (IFN-γ) release assays (IGRA) are currently the two preferred diagnostic tools for detecting TB infection. TST uses Tuberculin PPD RT 23 intradermally, which is affordable and easily performed.^7^ TST is currently the most frequently used TB infection test in Vietnam. However, TST has low sensitivity and specificity, particularly in people who are immunocompromised or have been BCG vaccinated, and people must return to a health facility to have the induration on their arm interpreted. IGRAs are more expensive and technically more complex, requiring phlebotomy, trained staff and laboratory equipment. Neither method is ideal for accurate and prompt diagnosis of TB infection in a community setting.^8^ Therefore, it is critical to develop new test methods to achieve the public health goal of preventing progression from TB infection to active TB and thereby reducing transmission.

Cy-Tb (Serum Institute of India, Pune, India) is an *M. tuberculosis* antigen-based skin test that was endorsed by the WHO in 2022.^9^ The test uses *M. tuberculosis*-specific antigens (rdESAT-6 and rCFP-10), which are injected intradermally, providing results after 48–72 hours, similar to the TST. However, a recent study indicated the superior performance of Cy-Tb compared with TST, with results in line with the QuantiFERON-TB Gold Plus (QFT-Plus) assay (QIAGEN, Venlo, The Netherlands).^10^

Simplified versions of IGRAs are emerging,^11^ including the lateral flow Standard F TB assay (SD Biosensor, Gyeonggi-do, Republic of Korea). Standard F TB has fewer manual processing steps than current-generation IGRAs. After incubation for 16–24 hours, the results will be available in 15 min. Following the stimulation with ESAT-6 and CFP-10 antigens, the IFN-γ production in whole blood is measured by a fluorescent immunoassay.^12^ A recent clinical trial comparing the sensitivity and specificity of the Standard F TB versus QIAreach QuantiFERON-TB (QIAGEN, Venlo, The Netherlands), using TST as a reference standard, showed that the Standard F TB has 88.9% sensitivity and 92.5% specificity for TB infection diagnosis.^13^ However, given the limited accuracy of TST in diagnosing TB infections, further studies comparing to the current gold standard are warranted. Compared with QFT-Plus, the Standard F TB assay may offer important advantages, such as being able to be implemented in low-resource settings, and reducing the financial burden on health systems and people with TB. The Standard F TB assay is currently undergoing Expert Review Panel for Diagnostics and WHO review.^14^

Currently, there are no studies comparing the accuracy of Standard F TB versus QFT-Plus. In addition, there are also no head-to-head comparisons of the sensitivity and specificity of Cy-Tb and Standard F TB. The studies comparing the accuracy of Cy-Tb test versus QFT-Plus have been conducted in Spain and South Africa,^9 15^ whereas there are no studies in a high TB burden, Asian population. This study was designed to address these knowledge gaps.

### Aims

#### Main objective

The main objective is to evaluate the accuracy of the Cy-Tb skin test and the Standard F TB assay versus the QFT-Plus assay as diagnostic methods of TB infection in Vietnam, a high TB burden setting.

#### Specific objectives

*Objective 1*: to verify the sensitivity of Cy-Tb and Standard F TB for TB infection diagnosis among adults with microbiologically confirmed pulmonary TB, using QFT-Plus test as a reference standard.

*Objective 2*: to evaluate the specificity and sensitivity of the Cy-Tb and Standard F TB tests, compared with QFT-Plus for TB infection diagnosis.

## Methods and analysis

### Study design

This diagnostic accuracy study will employ a cross-sectional study design and will be conducted from April 2024 to December 2024.

### Study population

For objective 1, the study will enrol 100 eligible participants with microbiologically confirmed pulmonary TB at the National Lung Hospital, Hanoi, Vietnam for the purpose of verifying the sensitivity of (1) Cy-Tb and (2) Standard F TB for TB infection diagnosis. For objective 2, to evaluate the sensitivity and specificity of the Cy-Tb and Standard F TB tests for TB infection diagnosis, the study will recruit 250 eligible participants, including 200 household contacts of people with pulmonary TB and 50 people with known negative IGRA tests who report no history of exposure to TB.

*Group 1*: people with microbiologically confirmed pulmonary TB. The sensitivity of the Cy-Tb test and Standard F TB assay to diagnose TB infection will be confirmed in this group.

*Group 2*: household contacts of persons with bacteriologically confirmed, pulmonary TB. The sensitivity and specificity of the Cy-Tb test and Standard F TB assay to diagnose TB infection will be verified in this group.

*Group 3*: persons with known negative IGRA tests among those at low risk of TB infection. The specificity of Cy-Tb and Standard F TB for TB infection testing will be estimated using this group.

#### Inclusion criteria

##### All participants

Participants above 18 years of age, able to provide informed consents and have no current plans to relocate outside the designated area for the duration of the study will be included in the study.

###### Group 1 only

- People with microbiologically confirmed pulmonary TB disease (either drug-susceptible TB or drug-resistant TB) via Xpert MTB/RIF Ultra (Cepheid, Sunnyvale, California) who also have an abnormal chest X-ray (CXR) result.*

*To reduce the false positive rate of Xpert Ultra assays.

###### Group 2 only

- People without symptoms of active TB disease who are household contacts* of people with new microbiologically confirmed pulmonary TB who initiated treatment in Ha Noi, Vietnam.
- Have a normal CXR result.

*Household contacts: of a person with TB are defined as members who live under the same roof as the person with contagious pulmonary TB or who meet the following conditions^4^:

- *Sleeping under the same roof or sharing a kitchen space as TB-affected persons at least one night/week for3 monthsbefore the person was diagnosed with TB*.
- *Staying under the same roof with TB-affected persons for at least1 hour/day and continuously5 days/week for3 monthsbefore the person was diagnosed with TB*.

###### Group 3 only

- Known past negative IGRA test results among those at low risk for TB infection.
- No known and/or reported history of contact or exposure to either TB disease or *M. tuberculosis* bacteria.
- Have a normal CXR.

### Exclusion criteria

Participants will be excluded from the study if they decline to give informed consent to participate in the study or if they are hypersensitive or contraindicated to TST or IGRA.

#### Groups 2 and 3

- Presumed TB disease with symptoms (cough, fever, night sweats, unintentional weight loss) and/or an abnormal CXR result suggestive of TB disease.
- Diagnosed with TB (microbiologically or clinically confirmed) in all forms or report having taken treatment for TB disease.
- History of TB infection (self-record or documented).

### Sample size

Sample size calculations used estimates from previously published studies on the sensitivity and specificity of Cy-Tb and Standard F TB,^10 13^ incorporating the prevalence of the disease. These calculations were based on the sample size calculation formula for sensitivity and specificity.^16^

#### Group 1

We assumed a true sensitivity of the Cy-Tb test and Standard F TB assay of 75.8% and 88.9%, respectively,^13^,^17^ with a precision of 0.1 for the related 95% CI and drop-out rate (lost to follow-up (LTFU)) of 10%. The smallest sample size to evaluate the sensitivity and specificity of the Cy-Tb test and Standard F TB assay in the group are 71 participants and 38 participants, respectively.

#### Group 2

We assumed a proportion of TB infection among group 2 of 50%,^18^ a true sensitivity of the Cy-Tb test and Standard F TB assay of 75.8% and 88.9% respectively,^13 17^ with a precision of 0.1 for the related 95% CI and drop-out rate (LTFU) of 10%. The smallest sample size to evaluate the sensitivity and specificity of the Cy-Tb test and Standard F TB assay in the group is 158 participants and 85 participants, respectively.

#### Group 3

We assumed a proportion of TB infection among group 3 of 5%, a true specificity of the Cy-Tb test and Standard F TB assay of 98.1% and 92.5% respectively,^13 17^ with a precision of 0.1 for the related 95% CI and drop-out rate (LTFU) of 10%. The smallest sample size to evaluate the specificity of the Cy-Tb test and Standard F TB assay in the group is 8 participants and 29 participants, respectively.

### Laboratory procedures

**Figure 1:**
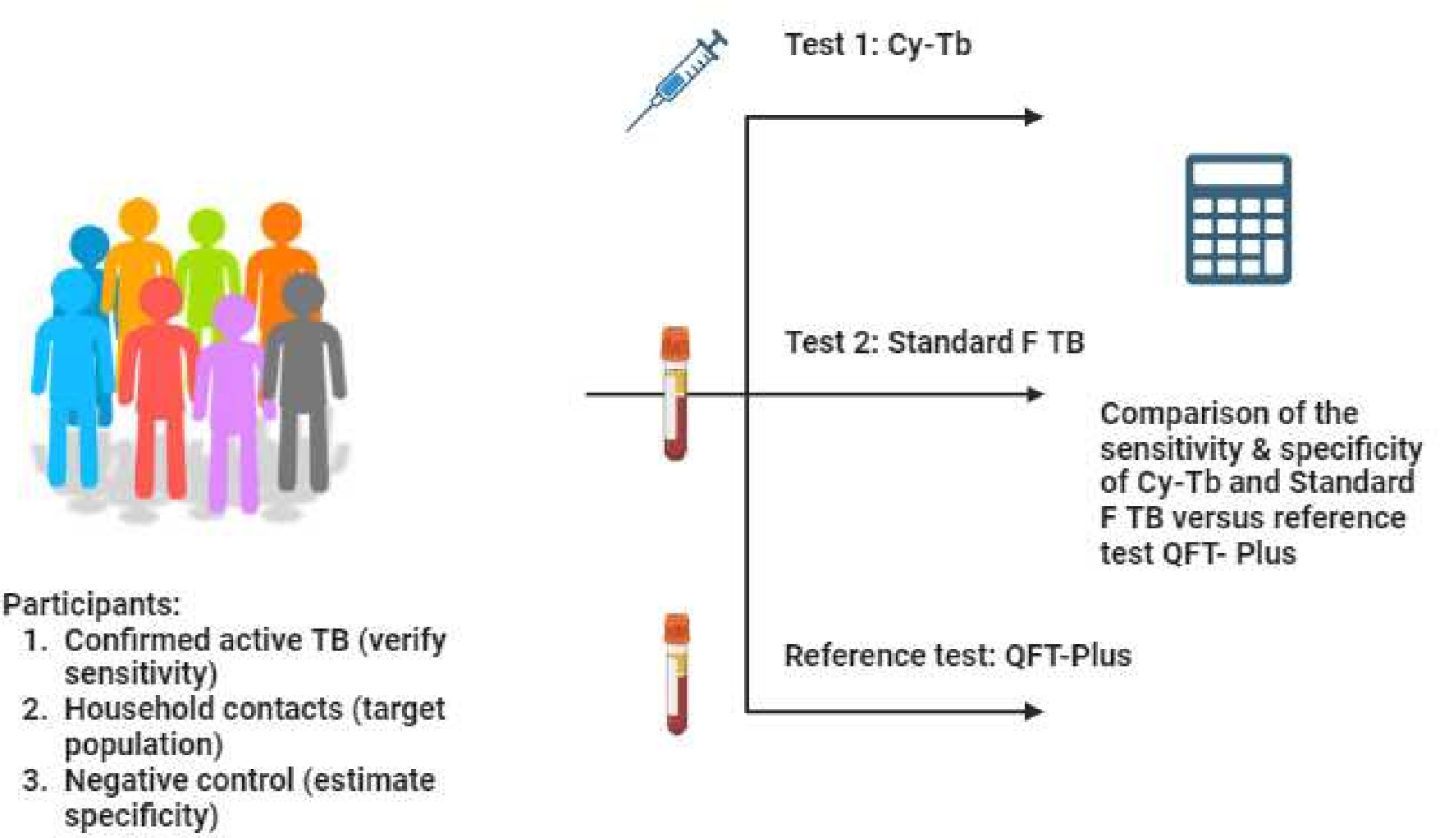
Diagnostic accuracy study overview. QFT-Plus, QuantiFERON-TB Gold Plus; Standard F TB, STANDARD F TB-Feron FIA; TB, tuberculosis.

After signing the Informed Consent Form (ICF) and undergoing eligibility assessment for study participation (including CXR, Xpert MTB/RIF Ultra and TB symptoms checklist), participants will undergo a baseline visit, followed by blood collection for Standard F TB and QFT tests. Subsequently, all participants will receive an injection of Cy-Tb (figure 1).

The CXR will be assessed by a single radiologist in the imaging department according to clinical routine and will be done prospectively and directly. The radiologist will be blinded to the Xpert MTB/RIF Ultra results.

10 mL of blood will be collected to three Standard F TB Tubes (Nil, TB Ag and the mitogen tube, 1 mL each tube) and four QFT-Plus tubes (Nil; TB1; TB2; Mitogen) for each participant, whereby the tubes will be incubated at the temperature of 37°C for a period of 16–24 hours. Standard F TB will be processed using the STANDARD F2400 equipment (SD Biosensor), an automated device. The ELISA test will be performed on QFT-Plus tubes. All tests will be processed and performed at the Vietnam National Reference Lab (VNRL)—National Lung Hospital. After being collected at the outpatient department at the same hospital, study samples will be transported to VNRL within 2 hours while still fresh. Lab personnel interpreting Standard F TB test results will be blinded to all other test results.

After blood collection, all participants will receive an intradermal injection of Cy-Tb and the results will be read after 48–72 hours. A solitary test dose of 0.1 mL comprises 0.05 µg each of rdESAT-6 and rCFP-10. These components elicit a delayed-type hypersensitivity reaction mediated by T lymphocytes. In individuals with previous mycobacterial exposure, this reaction results in induration at the injection site typically appearing within 48–72 hours, similar to the TST.^9^

In order to ensure the blinding of Standard F TB results to the reference test (QFT-Plus) and comparator test (Cy-Tb) and to the result of the microbiological reference standard including Xpert Ultra, different operators will be assigned and will be instructed to record results independently of other test results. Cy-Tb will be interpreted by a trained study nurse in the outpatient department.

### Safety

Adverse reactions to intradermal injections will be recorded and managed by the treating clinicians according to local treatment guidelines or standard algorithms. Guidance on the management of specific adverse reactions will be provided in a clinical management guide. The most common side effects of Cy-Tb are mild reactions at the injection site, for instance, itching and pain (23.7%–53.1%).^19^

### Endpoints

#### Data collection

Data on age, sex, demographics (occupation, residence, education level, living situation) body mass index (BMI), TB symptoms, TB exposure and previous TB disease or TB infection, and comorbidities (diabetes, immunodeficiency status, organ transplantation, etc) will be acquired through a preformed questionnaire during study visits. These data will be self-reported by participants and supplemented, where available, from the national TB registry and medical records. To assess immunodeficiency status, we will conduct thorough evaluations of participants’ medical history, including any known immunocompromising conditions such as HIV/AIDS, autoimmune diseases, malignancies or history of organ transplantation as well as the use of immunosuppressive medications like biologics, infliximab or checkpoint inhibitors. The questionnaire will be administered to participants through face-to-face interviews conducted by trained research personnel in a private and confidential setting at the Vietnam National Lung Hospital.

#### Endpoints

*Primaryendpoint*: point estimate of sensitivity with 95% CI of the Cy-Tb test and Standard F TB assay.

*Secondaryendpoint*: point estimates of sensitivity and specificity with 95% CI of the Cy-Tb test and Standard F TB assay.

### Data analysis plan

Points estimates of sensitivity and specificity with 95% CI will be calculated based on Wilson score methods to accommodate for the loss of coverage in small samples. The Cy-Tb and Standard F TB test index accuracy will be evaluated to fit with the case definition and results compared with the QFT-Plus reference standard test results presented in table 1.

**Table 1:** The case definition and results

	Definitions			
Test results		Tuberculosis infection	Not infected	Total
	Detected	True positive	False positive	True positive+false positive
	Not detected	False negative	True negative	False negative+true negative
	Total	True positive+false negative	False positive+true negative	Total

Concordant with the methods employed in a systematic review by WHO, we consider the rate of negative results to represent proxy specificity regardless of TB incidence and estimates.^20^ Differences in proportion of negative results between investigated tests and the reference test will be calculated. In addition, we will also calculate the rate of negative results on the Cy-Tb and Standard F TB tests among QFT-Plus negative participants.

Sensitivity is the percentage of participants with positive study tests among those with microbiologically confirmed TB (group 1).

Specificity is the percentage of participants with negative study tests among those at low risk for TB infection (group 3). We will also calculate the proportion of people with negative index tests among those at risk for TB infection (group 2).

The descriptive statistics will be calculated for the sample and the contingency table will be created to evaluate the diagnostic performance, including sensitivity, specificity and associated 95% CI for the Cy-Tb test and Standard F TB assay in comparison with the reference standard. The area under the curves, that is, areas under the receiver-operating characteristics curve will be calculated to estimate the comparative diagnostic accuracy of the Cy-Tb test and Standard F TB assay and QFT-Plus.

Multiple multivariate logistic regression models will be performed to adjust for confounders while analysing associations between participants characteristics and test accuracy. Subgroup analysis of test accuracy in different groups will be done with regard to gender, age, BMI, comorbidities, TB exposure, etc.

#### Statistical software

The data analysis will be performed using Stata V.17 or higher and Microsoft Excel V.2016 or higher for initial visualisation of the data.

### Significance

A simple, affordable and accurate test for TB infection can potentially make real changes in high-burden TB countries. The tests included in the study (Cy-Tb and Standard F) both have positive and negative attributes, and it is important to evaluate their performance in a high TB burden country such as Vietnam. If more people are correctly diagnosed with TB infection, preventative treatment can be provided and active cases can be averted, which in turn will decrease the number of people transmitting TB. The success of TB programmes is highly dependent on the active diagnosis and treatment of TB infections, which is not often performed in many high-endemic countries due to limited resources. The study will provide objective evidence for countries with high TB burden to issue new guidelines on the diagnosis of TB infection. However, a limitation is that the reference standard used in the study (QFT-Plus assay) also has suboptimal sensitivity, but is currently the best available test for the diagnosis of TB infection and is recommended by the WHO.

### Patient and public involvement

At this stage of the study, the tests under evaluation have not been implemented programmatically. Consequently, community participation in research will be limited. The project involves 350 individuals with TB infection or IGRA negative from the community. By involving participants in the study conducted in their community, the study aims to raise awareness about TB and the implications of early TB infection diagnosis within this community. Community members will be invited to any results dissemination meetings that are organised.

## Ethics and dissemination

### Ethics considerations

#### Regulatory and ethics approvals

The study will comply with the regulations on research ethics according to the Declaration of Helsinki and CIOMS guidelines as well as Vietnam’s national laws and regulations.

The study was granted ethical approvals from the Vietnam National Lung Hospital Institutional Review Board (65/23/CN-HDDD-BVPTU) and the Swedish Ethical Review Authority (Dnr 2023-04271-01).

#### Informed consent process

Research participants will be briefed on the content and significance and provided with ICFs, which must be signed to participate in the study. Any risks or benefits of participating in the study will be described in detail in the consent form.

#### Data management and protection

##### Data management

Research staff will record clinical data and laboratory results directly onto electronic case report forms (eCRFs) designed by Freundeskreis Für Internationale Tuberkulosehilfe e.V (FIT) in the ONA^21^ system. In cases where direct recording using the eCRFs is not possible, all data will be imported from paper CRF into ONA by site staff.

##### Missing data

Queries will be sent to study sites to correct erroneous data or complete missing information. We will not impute missing data.

##### Confidentiality and privacy

Participant information sheets will be stored in a locked cabinet and will be made accessible only to research staff. Data files, additional inputs and other profile information will be confidential. All participant information will be securely stored and encrypted and only pseudonymised data will be analysed, to ensure confidentiality and protect individual privacy.

### Safety considerations

Since this study will not use the test results to guide patient care, and considering the minimal risk associated with additional study procedures such as Cy-Tb and blood sample collection, the likelihood of a study participant experiencing serious adverse events (SAE) related to the investigational products is very low.

All AE-related information will be recorded. The SAE will be reported to FIT and all relevant authorities.

### Study benefits, costs or payments

#### Study benefits

There is no known direct benefit to participants for being in this study. The anticipated future benefit is improved diagnostics of TB infection. All participants who test positive on the QFT-Plus test will receive TB preventive treatment in accordance with the guidelines established by the Vietnam National Tuberculosis Programme.^7^

#### Cost or payments

Participants will receive the amount of 200 000 VND per study visit as reimbursement for study-related costs. There is no cost to participants or their healthcare provider if they participate in this study.

### Dissemination plan

Results will be published in an international journal and results only be presented at an aggregate group level without the risk of identifying individual study participants.
