# Supplementary material for: Study protocol: diagnostic accuracy study comparing Cy-Tb and STANDARD F TB-Feron FIA tests for tuberculosis infection diagnosis in Vietnam
Source: BMJ Open. 2024 Aug 9;14(8):e085614. doi: 10.1136/bmjopen-2024-085614 (PMC11331973; doi:10.1136/bmjopen-2024-085614)
Supplement: Supplementary file 1 [file bmjopen-2024-085614.reviewer_comments.pdf]

PEER REVIEW HISTORY

BMJ Open publishes all reviews undertaken for accepted manuscripts. Reviewers are asked to complete a checklist review form and are provided with free text boxes to elaborate on their assessment. These free text comments are reproduced below.

ARTICLE DETAILS

**TITLE (PROVISIONAL)**	Study protocol: Diagnostic accuracy study comparing Cy-Tb and STANDARD F TB-Feron FIA Tests for Tuberculosis Infection Diagnosis in Vietnam
**AUTHORS**	Nguyen, Han; Vo, Luan; Codlin, Andrew; Forse, Rachel; Wingfield, Tom; Sidney, Kristi; MacLean, Emily Lai-Ho; Creswell, Jacob; Kirubi, Beatrice; Davies Forsman, Lina

VERSION 1 - REVIEW

**REVIEWER NAME**	***Sabiiti, Wilber***
**REVIEWER AFFILIATION**	University of St Andrews, School of Medicine
**REVIEWER CONFLICT OF INTEREST**	None
**DATE REVIEW RETURNED**	24-Mar-2024

**GENERAL COMMENTS**	The authors have designed a study to evaluate two non-sputum new tests to diagnose TB infection. This is timely and a welcome step. However, I am concerns over the study that are highlighted below:<br><br>Tittle: The tittle 'Evaluating the accuracy of new tests for TB infection diagnosis' is quite misleading. It sounds as if it is covering all 'new TB tests' yet it is only evaluating two non-sputum tests.<br><br>Abstract: Undefined abbreviations Cy-TB and Standard F TB are use used in the abstract making it difficult to understand what they are.<br><br>Strengths and limitations: Conducting the study in a high burden setting is listed as one of the strengths. However, it is not clear that population of one country is sufficient to evaluate for tests that are likely to be affected by genetic polymorphisms present in different populations. As a limitation, the gold standard QFT-Plus is mentioned due to its limited sensitivity, couldn't the authors consider a composite gold standard that brings other parameters on board to increase accuracy of gold standard?<br><br>Specific comments<br>Line 67 Page 5: the statement is misleading vis-a-vis line 68 where TB is stated as 2nd to COVID-19.<br><br>Line 100 cites a clinical trail that used TST as a reference test. However TST is known to be a less accurate test for TB infection, so results obtained using it as a reference test should be treated with a pinch of salt.<br><br>Generally, the introduction does not give enough description of what Cy-TB and Standard F TB are and the principles by which they

	work. The authors ought to give adequate information about the tests for the readers to understand.<br><br>Line 126 - 140 referring to study population/groups. It is not clear how the authors calculated the sample of 100, 200 and 50. What is the hypothesis underlies these samples sizes and was any formula used to calculate the sample size? Lines 174 to 191 aren't clear enough as to explain how sample size was calculated.<br><br>Line 143: Inclusion criteria, is there any reason why age 18 and above was chosen? Was any maximum age beyond which participants will not be enrolled?<br><br>Line 192, what exactly is Cy-TB? Is it an antigen or antibody? The authors need to explain clearly what it is for the readers.<br><br>Line 199 - 200: Will samples be transported fresh or preserved in some way?<br><br>Line 217-221: Are there 95% CI targets to justify the accuracy of the test.<br><br>Line 249-251: It is difficult to achieve the intended significance because the results are going to evaluate the new tests using gold standard which they admit has limited sensitivity.

**REVIEWER NAME**	***Villa, Simone***
**REVIEWER AFFILIATION**	University of Milan
**REVIEWER CONFLICT OF INTEREST**	None
**DATE REVIEW RETURNED**	04-Apr-2024

**GENERAL COMMENTS**	This interesting protocol aims to evaluate the diagnostic accuracy of two new tests for TB infection diagnosis: the Cy-Tb and the Standard F TB assay. In general, the protocol is clear but I think it would benefit from the following improvements:<br><br>Lines 146-153: How chest radiography will be assessed? Are you using administrative data or will you assess it directly? As for the latter, are you blinding the person assessing the radiography to Xpert results? Are you using two persons to evaluate each radiography?<br>Line 193: Precise when the investigators expect to collect the 10mL of blood.<br>Line 213: Elaborate what "demographic" data you will collect.<br>Line 214: Define how you will assess, or define, the immunodeficiency status.<br>Line 215: How the questionnaire will be administered to participants<br>Lines 213-216: Is that information self-reported by the participants or assessed by study investigators, for example, by running additional tests (e.g., measuring glycemia for diabetes)?<br>Line 283: data will be anonymized before analysis, is it correct?<br><br>On another ethical note, are test-positive participants without active TB receiving preventive treatment?

VERSION 1 – AUTHOR RESPONSE

Reviewers' Comments:
Reviewer #1: Dr. Wilber Sabiiti, University of St Andrews
The authors have designed a study to evaluate two non-sputum new tests to diagnose TB infection. This is timely and a welcome step. However, I am concerns over the study that are highlighted below:
Response: Many thanks, Dr Sabiiti for your insightful and valuable feedback that we believe will greatly improve our manuscript. We hope we have answered all your queries below.
1. Tittle: The title 'Evaluating the accuracy of new tests for TB infection diagnosis' is quite misleading. It sounds as if it is covering all 'new TB tests' yet it is only evaluating two non-sputum tests.
Response:
Thank you for your comment. The title was updated as "Study protocol: Diagnostic accuracy study comparing Cy-Tb and STANDARD F TB-Feron FIA Tests for Tuberculosis Infection Diagnosis in Vietnam"
2. Abstract: Undefined abbreviations Cy-TB and Standard F TB are used in the abstract making it difficult to understand what they are.
Response:
Thank you for your feedback. In response, we have updated the terminology as follows: "Cy-Tb" and "Standard F-TB" are now referred to as The rdESAT-6 and rCFP-10 (Cy-Tb) Injection ("Cy-Tb"), a TB-specific antigen skin test, and STANDARD F TB-Feron FIA ("Standard F TB")- measuring Interferon-gamma (IFN-γ) by fluorescence immunoassay assay, respectively, as stated in lines 31-33.
3. Strengths and limitations: Conducting the study in a high-burden setting is listed as one of the strengths. However, it is not clear that the population of one country is sufficient to evaluate for tests that are likely to be affected by genetic polymorphisms present in different populations. As a limitation, the gold standard QFT-Plus is mentioned due to its limited sensitivity, couldn't the authors consider a composite gold standard that brings other parameters on board to increase the accuracy of the gold standard?
Response:
Thanks for this important comment. We completely agree and your point is one of the reasons we conduct this study in Vietnam, as the tests have previously been assessed in Spain1 and Chile2, but never in an Asian country and not in a head-to-head design. We advocate for future similarly designed studies in other countries with diverse ethnicities to enhance the generalizability of findings. In addressing the limited sensitivity of the gold standard QFT-Plus, our study design aims to mitigate this by specifically recruiting the two groups with the highest IGRA positivity rates: the confirmed TB group and the household contacts of people with pulmonary tuberculosis, who have bacteriologically confirmed TB, as outlined in section 2.2 of the study population. As IGRA is currently the gold standard for diagnosing TB infection and has been used as the reference standard in previous studies, we believe it is the best option available and will facilitate comparisons to other studies by using the same reference method.

Specific comments
4. Line 67 Page 5: the statement is misleading vis-a-vis line 68 where TB is stated as 2nd to COVID-19.
Response:
Thanks so much for pointing this out. To avoid confusion, we have deleted the sentence.
5. Line 100 cites a clinical trial that used TST as a reference test. However, TST is known to be a less accurate test for TB infection, so results obtained using it as a reference test should be treated with a pinch of salt.
Response:
We completely agree with your valid point and it is one of the reasons this study is needed. Currently, there are no studies directly comparing the Standard F TB-Feron FIA assay with the gold standard

QuantiFERON-TB Gold Plus; however, a study conducted in Chile2 did compare this assay with the TST. Although TST is recognized for its limited accuracy in diagnosing TB infection, it remains widely employed, particularly in resource-limited settings3. Therefore, our reference to this study is confined to the realm of research. Additionally, our study will directly compare the Standard F TB-Feron FIA assay with the gold standard test, QuantiFERON-TB Gold Plus. This approach enables a more precise evaluation of the sensitivity and specificity of the Standard F TB-Feron FIA assay. We have added the following sentence to clarify the issue:

Lines 100-105: A recent clinical trial comparing the sensitivity and specificity of the Standard F TB versus QIAreach QuantiFERON-TB (QIAGEN, Venlo, The Netherlands), using TST as a reference standard, showed that the Standard F TB has a high accuracy in TB infection diagnosis (88.9% sensitivity and 92.5% specificity) 13. However, given the limited accuracy of TST in diagnosing TB infections, further studies comparing to the current gold standard are warranted.

Line 110: Currently, there are no studies comparing the accuracy of Standard F TB versus QFT-Plus.

6. Generally, the introduction does not give enough description of what Cy-TB and Standard F TB are and the principles by which they work. The authors ought to give adequate information about the tests for the readers to understand.

Response:

We thank you for the opportunity to clarify our manuscript and have added the following sentences in the Introduction section, lines 91-103

Cy-Tb (Serum Institute of India Pvt. Ltd, Pune, India) is an M. tuberculosis antigen-based skin test that was endorsed by the WHO in 2022 9. The test uses MTB-specific antigens (rdESAT-6 and rCFP-10), which are injected intradermally, providing results after 48-72 hours, similar to the TST. However, a recent study indicated the superior performance of Cy-Tb compared to TST, with results in line with the QFT-Plus assay (QIAGEN, Venlo, The Netherlands) 10.

Simplified versions of IGRAs are emerging11, including the lateral flow Standard F TB assay (SD BIOSENSOR, INC, Gyeonggi-do, Republic of Korea). Standard F TB has fewer manual processing steps than current-generation IGRAs. After incubation for 16-24 hours, the results will be available in 15 minutes. Following the stimulation with ESAT-6 and CFP-10 antigens, the IFN-γ production in whole blood is measured by a fluorescent immunoassay 12. A recent clinical trial comparing the sensitivity and specificity of the Standard F TB versus QIAreach QuantiFERON-TB (QIAGEN, Venlo, The Netherlands), using TST as a reference standard, showed that the Standard F TB has 88.9% sensitivity and 92.5% specificity for TB infection diagnosis 13.

7. Line 126 - 140 referring to study population/groups. It is not clear how the authors calculated the sample of 100, 200 and 50. What is the hypothesis underlies these samples sizes and was any formula used to calculate the sample size? Lines 174 to 191 aren't clear enough as to explain how sample size was calculated.

Response:

The sample size calculations used estimates from previously published studies on the sensitivity and specificity of Cy-Tb and Standard F TB 1,2, incorporating the prevalence of the disease infection. These calculations were based on the sample size calculation formula for sensitivity and specificity. The formulation was from “Buderer NM. Statistical methodology: I. Incorporating the prevalence of disease into the sample size calculation for sensitivity and specificity. Academic emergency medicine: official journal of the Society for Academic Emergency Medicine 1996;3(9):895-900. doi: 10.1111/j.1553-2712.1996.tb03538.x”4.

We agree that this needs to be clarified and have added a detailed description of the calculations as supplementary information.

8. Line 143: Inclusion criteria, is there any reason why age 18 and above was chosen? Was any maximum age beyond which participants will not be enrolled?

Response:

The prevalence of TB infection is typically lower in individuals under 18 compared to those over 18 years old. In a systematic review of contact investigations involving children exposed to tuberculosis in Southeast Asia, it was found that the prevalence of infection among all children ranged from 24.4%

to 38.8%5.
Using the Burderer sample size formula, the TB infection rate among children in the household contacts group ranged from 24.4% to 38.8% (Prevalence = 24.4% - 38.8%). We assumed a true sensitivity of the Cy-Tb test and Standard F TB assay of 75.8% (SN)1 and 88.9% (SN)2, respectively, and a true specificity of the Cy-Tb test and Standard F TB assay of 98.1% (SP)1 and 92.5% (SP)2, respectively. We also considered a precision of 0.1 (L) for the related 95% CI (α=0.5%), and a drop-out rate (lost to follow-up- LTFU) of 10%1-2.
With a prevalence of 24.4%, the smallest sample sizes required to evaluate the sensitivity and specificity of the Cy-Tb test and Standard F TB assay in the group are 322 participants and 174 participants, respectively.
With a prevalence of 38.8%, the smallest sample sizes required to evaluate the sensitivity and specificity of the Cy-Tb test and Standard F TB assay in the group are 203 participants and 109 participants, respectively.
Hence, considering our current budget, we do not anticipate recruiting a sufficient number of children to conduct a meaningful age-stratified subgroup analysis.
There is no upper age limit for participant registration; individuals of all ages are eligible to participate in the study.
9. Line 192, what exactly is Cy-TB? Is it an antigen or antibody? The authors need to explain clearly what it is for the readers.
Response:
Cy-Tb (Serum Institute of India Pvt. Ltd, Pune, India) is an M. tuberculosis antigen-based skin test that was endorsed by the WHO in 2022 1. The test uses MTB-specific antigens (ESAT6 and CFP10) which stimulate a delayed-type hypersensitivity reaction mediated by T lymphocytes. In individuals with previous mycobacterium tuberculosis exposure, this reaction results in induration at the injection site typically appearing within 48 to 72 hours, similar to the TST.
We have clarified this in lines 213-218, as follows:
After blood collection, all participants will receive an intradermal injection of Cy-Tb and the results will be read after 48-72 hours. A single test dose of 0.1 mL comprises 0.05 µg each of rdESAT-6 and rCFP-10. These components elicit a delayed-type hypersensitivity reaction mediated by T lymphocytes. In individuals with previous mycobacterial exposure, this reaction results in induration at the injection site typically appearing within 48 to 72 hours, similar to the TST.
10. Line 199 - 200: Will samples be transported fresh or preserved in some way?
Response: After being collected at the Outpatient Department at the same hospital, study samples will be transported to the Vietnam National Reference Lab within 2 hours while still fresh.
This information has been added to lines 210-211.

11. Line 217-221: Are there 95% CI targets to justify the accuracy of the test.
Response: We will assess the diagnostic performance measurements, including sensitivity, specificity, and associated 95% confidence intervals for Cy-Tb and Standard F TB assay in comparison with the reference standard. If we employ a target below the lower limit of the 95% confidence interval, the investigator tests would fail to meet the acceptable accuracy criteria outlined in the target product profiles (TPP) mandated by the WHO for biomarker-based, non-sputum-based tests designed to detect TB infection6.
12. Line 249-251: It is difficult to achieve the intended significance because the results are going to evaluate the new tests using the gold standard which they admit has limited sensitivity.
Response:
We completely agree, and this has been added to the limitation section. Unfortunately, despite its limited sensitivity, the gold standard (QFT) is endorsed by the WHO as the best test available. If alternative tests were as sensitive and specific as QFT, they could provide more cost-effective testing options, particularly for low-resource countries and those with high TB burden. This would expand access to TB preventive treatment (TPT), ultimately reducing the global TB infection rate and advancing toward the goal of eliminating TB worldwide.

The limitation has been added in section 2.8 Significance as follows:
Lines 282- 285: However, a limitation is that the reference standard used in the study (QFT-Plus assay) also has suboptimal sensitivity, but is currently the best available test for the diagnosis of TB infection and is recommended by the World Health Organization (WHO).

Reviewer #2: Dr. Simone Villa, University of Milan
Comments to the Author:
This interesting protocol aims to evaluate the diagnostic accuracy of two new tests for TB infection diagnosis: the Cy-Tb and the Standard F TB assay. In general, the protocol is clear but I think it would benefit from the following improvements:
Response:
Thank you Dr Villa for your constructive and helpful feedback.
1. Lines 146-153: How chest radiography will be assessed? Are you using administrative data or will you assess it directly? As for the latter, are you blinding the person assessing the radiography to Xpert results? Are you using two persons to evaluate each radiography?
Response:
The chest radiographs will be assessed by a single radiologist in the imaging department according to clinical routine and will be done prospectively and directly. The radiologist will be blinded to the Xpert results. This information has been added to lines 201-203. Due to constraints in personnel, only one person will evaluate the radiography, as this is not the main outcome of the study.
2. Line 193: Precise when the investigators expect to collect the 10mL of blood.
Response:
Thank you for your comment. After signing the Informed Consent Form (ICF) and undergoing eligibility assessment for study participation, participants will undergo a baseline visit, followed by blood collection for Stand F TB and QFT tests. Subsequently, all participants will receive an injection of Cy-Tb.
This process is further elaborated in section 2.4, Laboratory Procedures from lines 197-218.
After signing the Informed Consent Form (ICF) and undergoing eligibility assessment for study participation (including CXR, Xpert MTB/RIF Ultra, and TB symptoms checklist), participants will undergo a baseline visit, followed by blood collection for Stand F TB and QFT tests. Subsequently, all participants will receive an injection of Cy-Tb.
The CXR will be assessed by a single radiologist in the imaging department according to clinical routine and will be done prospectively and directly. The radiologist will be blinded to the Xpert MTB/RIF Ultra results.
10 mL of blood will be collected to 3 Standard F TB Tubes (Nil, TB Ag, and the mitogen tube, 1 ml each tube) and 4 QFT-Plus tubes (Nil; TB1; TB2; Mitogen) for each participant, whereby the tubes will be incubated at the temperature of 37◦C for a period of 16 to 24 hours. Standard F TB will be processed using the STANDARD F2400 equipment (SD Biosensor), an automated device. The enzyme-linked immunoassay (ELISA) test will be performed on QFT-Plus tubes. All tests will be processed and performed at the Vietnam National Reference Lab (VNRL) - National Lung Hospital. After being collected at the Outpatient Department at the same hospital, study samples will be transported to VNRL within 2 hours while still fresh. Lab personnel interpreting Standard F TB test results will be blinded to all other test results.
After blood collection, all participants will receive an intradermal injection of Cy-Tb and the results will be read after 48-72 hours. A solitary test dose of 0.1 mL comprises 0.05 µg each of rdESAT-6 and rCFP-10. These components elicit a delayed-type hypersensitivity reaction mediated by T lymphocytes. In individuals with previous mycobacterial exposure, this reaction results in induration at the injection site typically appearing within 48 to 72 hours, similar to the TST 9.
3. Line 213: Elaborate what "demographic" data you will collect.
Response:
Thanks for the suggestion. We have added the following sentence at line 227 to clarify what demographics consist of.

Line 230-233: Data on age, sex, demographics (occupation, residence, education level, living situation) body mass index (BMI), TB symptoms, TB exposure and previous TB disease or TB infection, and comorbidities (diabetes, immunodeficiency status, organ transplantation, etc) will be acquired through a preformed questionnaire during study visits.
4. Line 214: Define how you will assess, or define, the immunodeficiency status.
Response:
Thank you for your comments
We have clarified this on lines 235-238: To assess immunodeficiency status, we will conduct thorough evaluations of participants' medical history, including any known immunocompromising conditions such as HIV/AIDS, autoimmune diseases, malignancies, or history of organ transplantation as well as the use of immunosuppressive medications like biologics, infliximab, or checkpoint inhibitors.
5. Line 215: How the questionnaire will be administered to participants
Response:
Thanks for your suggestion. We have added this information on lines 238-240: The questionnaire will be administered to participants through face-to-face interviews conducted by trained research personnel in a private and confidential setting at the Vietnam National Lung Hospital.
6. Lines 213-216: Is that information self-reported by the participants or assessed by study investigators, for example, by running additional tests (e.g., measuring glycemia for diabetes)?
Response:
Thanks for your comments. We have clarified this on lines 233-235: These data will be self-reported by participants and supplemented, where available, from the national TB registry and medical records.
7. Line 283: data will be anonymized before analysis, is it correct?
Response:
Yes, that is correct. We have added a sentence to clarify.
Lines 310-311:
All participant information will be securely stored and encrypted and only pseudonymized data will be analyzed, to ensure confidentiality and protect individual privacy.
8. On another ethical note, are test-positive participants without active TB receiving preventive treatment?
Response: All participants who test positive on the QFT-Plus test will receive TB preventive therapy in accordance with the guidelines established by the Vietnam National Tuberculosis Program. This was included in the study benefits section on lines 319-321.

Reviewer: 1
Competing interests of Reviewer: None

Reviewer: 2
Competing interests of Reviewer: None
References:
1. Ruhwald M, Aggerbeck H, Gallardo RV, et al. Safety and efficacy of the C-Tb skin test to diagnose Mycobacterium tuberculosis infection, compared with an interferon γ release assay and the tuberculin skin test: a phase 3, double-blind, randomised, controlled trial. The Lancet Respiratory Medicine 2017;5(4):259-68. doi: https://doi.org/10.1016/S2213-2600(16)30436-2
2. Saint-Pierre G, Conei D, Cantillana P, et al. Comparison of Two Tuberculosis Infection Tests in a South American Tertiary Hospital: STANDARD F TB-Feron FIA vs. QIAreach(TM) QuantiFERON-TB. Diagnostics (Basel, Switzerland) 2023;13(6) doi: 10.3390/diagnostics13061162.
3. Faust L, Ruhwald M, Schumacher S, Pai M. How are high-burden countries implementing policies and tools for latent tuberculosis infection? A survey of current practices and barriers. Health Sci Rep. 2020 May 3;3(2):e158. doi: 10.1002/hsr2.158. PMID: 32373716; PMCID: PMC7196590.
4. Buderer NM. Statistical methodology: I. Incorporating the prevalence of disease into the sample size calculation for sensitivity and specificity. Academic emergency medicine: official journal of the

Society for Academic Emergency Medicine 1996;3(9):895-900. doi: 10.1111/j.1553-2712.1996.tb03538.x
5. Rina Triasih, Merrin Rutherford, Trisasi Lestari, Adi Utarini, Colin F. Robertson, Stephen M. Graham, "Contact Investigation of Children Exposed to Tuberculosis in South East Asia: A Systematic Review", Journal of Tropical Medicine, vol. 2012, Article ID 301808, 6 pages, 2012. https://doi.org/10.1155/2012/301808
6. WHO, High priority target product profiles for new tuberculosis diagnostics: report of a consensus meeting, 28-29 April 2014, Geneva, Switzerland, available at: https://iris.who.int/bitstream/handle/10665/135617/WHO_HTM_TB_2014.18_eng.pdf?sequence=1
